# Supplementary material for: Identification of photoperiod-induced specific miRNAs in the adrenal glands of Sunite sheep (Ovis aries)
Source: Front Vet Sci. 2022 Jul 22;9:888207. doi: 10.3389/fvets.2022.888207 (PMC9354845; doi:10.3389/fvets.2022.888207)
Supplement: Supplementary file 1 [file Data_Sheet_1.ZIP › Supplementary file/miRDeep2 file.zip!/miRDeep2 file/Know_Structure/SPLP42A3/oar-mir-30b.pdf]

miRBase precursor : oar-mir-30b
Total read count : 12336
oar-miR-30b read count : 12191
remaining reads : 145

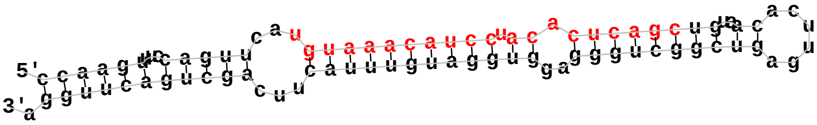

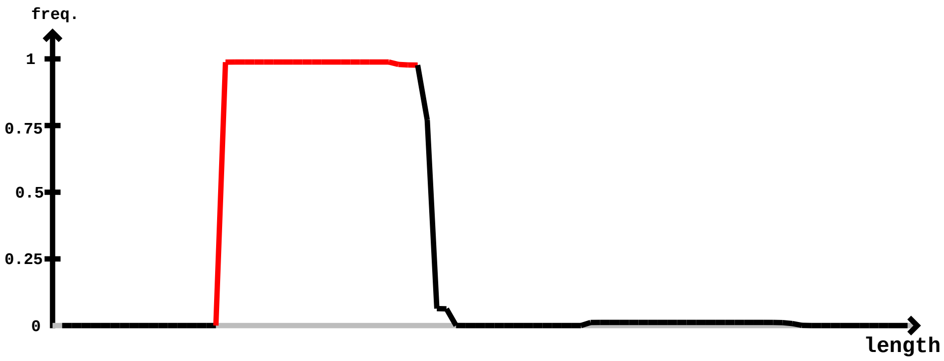


oar-miR-30b

```
5'- ccaaguuuccaguucauguaaacauccuacacucagcuguaacacuugagucggcugggagguggauguuuacuucagcugacuugga   -3'    exp
    ((((((...(((((...((((((((((.((.((((((((..((......))))))))))..))))))))))))...))))))))))).    reads      mm         sample
    ................uguaaacauccuacacuU......................................................    16         1          seq
    ................uguaaacauccuacacuc......................................................    95         0          seq
    ................uguaaacauccuacacuca.....................................................    25         0          seq
    ................uguaaacauccuacacucag....................................................    7          0          seq
    ................uguaaacauccuacacucagc...................................................    2536       0          seq
    ................uguaaacauccuacacucagcu..................................................    8722       0          seq
    ................uguaaacauccuacacucagcug.................................................    12         0          seq
    ................uguaaacauccuacacucagcugu................................................    770        0          seq
    ................uguaaacauccuacacucagcugua...............................................    2          0          seq
    .................guaaacauccuacacucagc...................................................    3          0          seq
    .................guaaacauccuacacucagcu..................................................    2          0          seq
    .................guaaacauccuacacucagcugu................................................    1          0          seq
    ......................................................cugggagguggauguuuac...............    1          0          seq
    ......................................................cugggagguggauguuuacu..............    8          0          seq
    ......................................................cugggagguggauguuuacuu.............    43         0          seq
    ......................................................cugggagguggauguuuacuuc............    81         0          seq
    ......................................................cugggagguggauguuuacuuca...........    9          0          seq
    .......................................................ugggagguggauguuuacuu.............    1          0          seq
    .......................................................ugggagguggauguuuacuuc............    1          0          seq
    .......................................................ugggagguggauguuuacuuca...........    1          0          seq
```
